# Postoperative symptom cluster classification and its association with health-related quality of life in knee osteoarthritis patients after total knee arthroplasty: An observational study

**DOI:** 10.1097/MD.0000000000049797

**Published:** 2026-07-17

**Authors:** Lei Zhou, Xingren Chen, Qingxi Zhang, Tong Chen

**Affiliations:** a Department of Orthopedics, Beijing Chaoyang Hospital, Capital Medical University, Beijing, People’s Republic of China.

**Keywords:** knee osteoarthritis, latent class analysis, quality of life, symptom clusters, total knee arthroplasty

## Abstract

Total knee arthroplasty (TKA) is an effective surgical treatment for advanced knee osteoarthritis (KOA). However, the classification of postoperative symptom clusters and their impact on quality of life (QoL) remain underexplored. This study aims to categorize symptom clusters in KOA patients after TKA using latent class analysis (LCA) and to analyze the relationship between symptom clusters and QoL. Standardized questionnaires were used to assess postoperative symptoms, including pain, swelling, anxiety, depression, and sleep disorders. LCA was employed to classify patients into high-symptom (C1), low-symptom (C2), and high-swelling (C3) groups. Multivariate unordered multinomial logistic regression was applied to analyze the association between symptom clusters and clinical variables. Differences in SF-12 health survey total scores and dimensional scores among symptom clusters were also compared. A total of 365 KOA patients who underwent TKA were analyzed (from 389 valid respondents; valid response rate 93.8%). LCA identified 3 distinct symptom clusters: C1 (high-symptom group, n = 59; 16.2%), C2 (low-symptom group, n = 186; 51.0%), and C3 (high-swelling group, n = 120; 32.9%). Key factors differentiating the high-symptom group from the low-symptom group included BMI, first joint surgery, and swelling severity (*P* < .05). On the SF-12, the total score of C1 (50.38 ± 12.96) was significantly lower than that of C2 (56.63 ± 9.56, *P* = .003) and C3 (57.42 ± 10.85, *P* = .004). The mental dimension score was significantly lower in C1 (55.22 ± 13.72) than in C2 (63.17 ± 11.33, *P* < .001) and C3 (63.61 ± 12.23, *P *< .001). Postoperative symptom cluster classification is an effective tool for assessing the symptom profile of KOA patients after TKA. Significant differences in QoL were found across symptom clusters, with the high-symptom and high-swelling groups showing lower quality of life, particularly in mental health. This study provides data support for individualized management of postoperative symptoms in KOA patients, highlighting the importance of comprehensive symptom assessment in postoperative care.

## 1. Introduction

Knee osteoarthritis (KOA) is a prevalent chronic degenerative joint disorder characterized by progressive cartilage degradation, synovial inflammation, and biomechanical instability, leading to persistent pain, articular stiffness, and functional limitation.^[1,2]^ This condition poses a major public health burden, particularly among older adults, ranking as the foremost cause of disability globally.^[3,4]^ Recent epidemiological studies estimate the global prevalence to exceed 250 million individuals, with a twofold higher incidence rate in females and marked acceleration after age 60.^[5]^ Pathophysiological progression involves the interplay of biomechanical stressors, metabolic dysregulation, and inflammatory mediators, with risk factors including obesity, joint trauma, and metabolic syndrome.^[6]^ Clinical management of KOA demonstrates heterogeneous treatment outcomes. While nonsurgical interventions such as pharmacotherapy and physical therapy provide symptomatic relief for early-stage disease, total knee arthroplasty (TKA) remains the gold-standard treatment for end-stage osteoarthritis, achieving significant pain reduction (Visual Analogue Scale score improvement > 50%) and functional recovery (6-minute walk distance increase > 30%) in 85% to 90% of patients at 5-year follow-up.^[7,8]^ Longitudinal studies have demonstrated sustained clinical benefits with 80% patient satisfaction rates maintained through 15 to 20 years post-surgery.^[9,10]^

Despite these successes, postoperative residual symptoms remain a critical clinical challenge. Meding et al^[11]^ demonstrated that gait speed and quadriceps strength in TKA recipients achieve only 60% to 70% of age-matched healthy controls’ levels at 12-month follow-up. Similarly, Berghmans et al^[12]^ reported that 31% of young (<50) patients continued to experience chronic pain (VAS ≥ 3/10) and activity limitations up to 7 years postoperatively. These findings underscore the necessity of multidimensional outcome assessment beyond traditional pain and functional metrics in TKA rehabilitation. Current research paradigms primarily adopt univariate symptom analysis, focusing on isolated postoperative manifestations such as pain, swelling, and range-of-motion deficit. This approach fails to capture the synergistic effects of symptom complexes that significantly impact recovery trajectories. Emerging evidence suggests that psychosomatic symptoms (anxiety, depression, sleep disturbances) and cognitive impairments frequently co-occur with physical symptoms, creating vicious cycles that exacerbate functional decline and reduce quality of life (QoL).^[13-15]^ The clustered nature of postoperative symptoms has been increasingly recognized in oncology and cardiology populations, yet remains underexplored in orthopedic surgery.

Latent class analysis (LCA) emerges as a promising methodological framework for deciphering symptom clusters in KOA post-TKA populations.^[16]^ As a probabilistic modeling technique, LCA identifies hidden subgroups (latent classes) through multivariate pattern recognition of observed symptoms. Recent applications in chronic pain management demonstrated that symptom cluster classification improves prediction accuracy of treatment response by 22% to 35% compared to conventional analysis methods.^[17]^ By applying LCA to postoperative symptoms, it is possible to classify patients into groups with similar symptom profiles, revealing hidden patterns that are not immediately apparent through traditional analysis. This innovative approach provides valuable insights into the complex interplay of symptoms and their effects on recovery and quality of life.

The primary aim of this study is to categorize postoperative symptom clusters in KOA patients following TKA and to evaluate their association with QoL. By employing LCA, the study seeks to identify distinct groups of symptoms – such as pain, swelling, anxiety, depression, and sleep disorders – that commonly occur together in post-TKA patients. These symptom clusters will be analyzed to determine how they correlate with clinical outcomes, including physical function and mental health, and to assess their collective impact on patients’ overall QoL during the postoperative period.

## 2. Materials and methods

### 2.1. Study design and participants

This was a prospective, single-center observational study conducted at the Department of Orthopedics, Beijing Chaoyang Hospital, Capital Medical University, and reported in accordance with the STROBE guideline for observational studies. Consecutive patients undergoing primary TKA for advanced KOA were enrolled and assessed at 3 time points: presurgery (baseline), 6 weeks post-surgery, and 12 weeks post-surgery. The present analysis of postoperative symptom clusters and their association with health-related quality of life was based on data collected at the 12-week postoperative assessment.

### 2.2. Inclusion and exclusion criteria

#### 2.1.1. Inclusion criteria

Adults aged 45 to 80 years with a clinical diagnosis of KOA, confirmed by radiographic evidence, were included. Patients who had undergone TKA as a treatment for severe KOA and were willing to participate in the study and provide informed consent were also included. Exclusion criteria included a history of previous knee surgeries or joint replacements, and cognitive impairment or severe psychiatric conditions that could interfere with the completion of assessments.

#### 2.1.2. Exclusion criteria

Patients with active infections or other major systemic conditions (e.g., uncontrolled diabetes, cardiovascular disease) that could affect recovery were excluded. Also excluded were patients with contraindications for TKA or who were expected to undergo revision surgery during the study period, pregnant or lactating women, and patients unable to complete self-reported questionnaires due to language barriers or physical impairments.

An a priori power analysis indicated that a minimum of 365 participants would provide 80% statistical power to detect clinically meaningful differences in health-related quality of life across symptom clusters at a 2-sided significance level of 0.05, based on effect sizes reported in previous studies. A total of 365 participants with complete cluster-defining data were available and analyzed, satisfying this target.

### 2.3. Ethical considerations

This prospective observational study was reviewed and approved by the Ethics Review Committee of Beijing Chaoyang Hospital, Capital Medical University (approval number: Beijing Chaoyang Hospital Medical Ethics Review 2024, Document No. 036; approval date: April 12,2024). The study was conducted in accordance with the Declaration of Helsinki and applicable national regulations and Good Clinical Practice standards. Before enrollment, all participants received a detailed explanation of the study objectives, procedures, potential risks and benefits, the voluntary nature of participation, and their right to withdraw at any time without affecting their medical care. Written informed consent was obtained from every participant. All data were anonymized and stored securely in compliance with the applicable ethical and data-protection regulations.

### 2.4. Data collection

Data collection took place at 3 key time points: presurgery (baseline), 6 weeks post-surgery, and 12 weeks post-surgery. These time points were chosen to capture the immediate postoperative recovery phase (6 weeks) and early recovery progression (12 weeks), allowing for an assessment of changes in symptoms and quality of life over time. The data collection process involved direct interaction with participants at each of these time points, where they completed a series of self-reported questionnaires. Additionally, clinical and demographic data were obtained through medical records and patient interviews.

### 2.5. Standardized questionnaires used to assess postoperative symptoms

*Pain (Visual Analog Scale - VAS):* Pain severity was assessed using the Visual Analog Scale (VAS),^[18]^ a widely used tool that measures pain intensity on a 10 cm line, with 1 end labeled “no pain” and the other end labeled “worst possible pain.” Patients were asked to mark the point on the line that best represented their pain intensity. The VAS score ranges from 0 (no pain) to 10 (worst imaginable pain).

*Swelling:* Swelling was evaluated through both patient self-report and clinician assessment. A Likert scale (ranging from 0 to 4) was used to rate the severity of swelling in the knee joint, with 0 representing no swelling and 4 representing severe swelling.^[19]^ This method allows for both subjective and objective evaluation of swelling, a common and significant postoperative symptom.

*Anxiety and depression (Hospital Anxiety and Depression Scale - HADS):* The Hospital Anxiety and Depression Scale (HADS) was used to assess the psychological impact of surgery, focusing on anxiety and depression symptoms.^[20]^ This scale consists of 14 items, 7 addressing anxiety and 7 addressing depression. Each item is scored on a 4-point Likert scale (ranging from 0 to 3), with higher scores indicating greater severity of anxiety or depression.

*Sleep disorders (Pittsburgh Sleep Quality Index - PSQI):* The Pittsburgh Sleep Quality Index (PSQI) was utilized to assess sleep quality and disturbances.^[21]^ This 19-item questionnaire evaluates sleep duration, disturbances, sleep latency, and overall sleep quality. Each item is scored on a scale of 0 to 3, with higher scores indicating worse sleep quality.

In addition to the symptom-specific questionnaires, we collected the following demographic and clinical characteristics to identify factors influencing symptom clusters and recovery outcomes: participants’ age at surgery (categorized as 45–59 years or ≥60 years), gender (male/female), body mass index (BMI; calculated from height and weight, classified into <24.0, 24.0–27.9, or ≥28.0 kg/m^2^), disease duration (≤5 years vs >5 years), number of prior joint surgeries (coded as 1 for first TKA and 2 for ≥2 surgeries), occupation (employed/self-employed, farmer, unemployed/retired, or other), marital status (married, divorced/widowed, or other), and family monthly income (stratified into 4 groups: <2000 Renminbi [Chinese Yuan; RMB], 2000–3999 RMB, 4000–5999 RMB, or ≥6000 RMB).

### 2.6. Latent class analysis

LCA was applied to identify latent symptom clusters in post-TKA patients by analyzing patterns of responses across multiple observed variables. This statistical method clusters individuals into unobserved subgroups based on shared characteristics, particularly useful for revealing hidden structures in complex clinical data. Our LCA model incorporated both postoperative symptoms and clinical covariates: symptom measures included pain (VAS), swelling (Likert scale), anxiety/depression (HADS), and sleep quality (PSQI); clinical characteristics consisted of age (<60 vs ≥60 years), gender, BMI (<24.0, 24.0–27.9, or ≥28.0 kg/m^2^), disease duration (≤5 vs >5 years), prior joint surgeries (first TKA coded as 1, ≥2 surgeries as 2), occupation, and marital status. To determine the optimal number of classes, model selection relied on multiple fit indices: Akaike Information Criterion (AIC) and Bayesian Information Criterion (BIC) balanced goodness-of-fit and complexity, with lower values indicating superior models; Sample-Adjusted BIC (aBIC) was prioritized for smaller sample sizes. Entropy (>0.85 considered excellent class separation) and likelihood ratio tests (Lo–Mendell–Rubin LMRT/bootstrap BLRT with *P* < .05 significance) validated model stability.

### 2.7. Statistical analysis

Statistical analyses included a descriptive summary of demographics (age, gender, BMI, marital status, occupation) and clinical variables (disease duration, prior surgeries) with continuous variables presented as mean ± standard deviation and categorical variables as frequencies/percentages. Symptom severity was quantified using standardized scores: VAS pain (0–100 mm), HADS anxiety/depression (0–21 points), and PSQI (0–21 points). A multinomial logistic regression model assessed associations between latent symptom clusters (low symptom: reference, high symptom, high swelling) and clinical covariates (age, gender, BMI category, disease duration, prior surgeries). Results were reported as odds ratios (ORs; 95% confidence intervals [CIs]) with *P* < .05 indicating statistical significance.

### 2.8. Outcome measures

Statistical analyses were conducted using SPSS statistics version 27.0 (IBM Corp., Armonk) and R version 4.3.1 (R Foundation for Statistical Computing, Vienna, Austria). Demographic variables (age, gender, BMI, marital status, occupation) and clinical parameters (disease duration, prior surgeries) were coded as categorical variables with specified cutoffs: BMI < 24.0 kg/m^2^, disease duration ≤ 5 years, and prior surgeries categorized as first TKA = 1 or ≥2 surgeries = 2. Symptom measures included continuous variables: VAS pain (0–100 mm), HADS anxiety/depression (0–21 points), and PSQI sleep quality (0–21 points). LCA identified 3 symptom clusters – C1 (high-symptom group), C2 (low-symptom group, used as the reference category), and C3 (high-swelling group) – based on VAS pain, swelling (Likert scale), HADS-anxiety, HADS-depression, and PSQI, fitted via the expectation–maximization algorithm. Model selection was guided by the AIC, BIC, and aBIC, together with entropy (a value approaching or exceeding 0.80 indicating adequate class separation) and the LMRT and BLRT. Model validity was assessed using the Hosmer–Lemeshow goodness-of-fit test (*P* > .10) and split-sample validation (70% training vs 30% validation). Missing data were handled by multiple imputation (5 iterations).

## 3. Results

### 3.1. Latent class analysis characteristics of postoperative dymptom clusters in KOA patients after TKA

**Figure 1.**
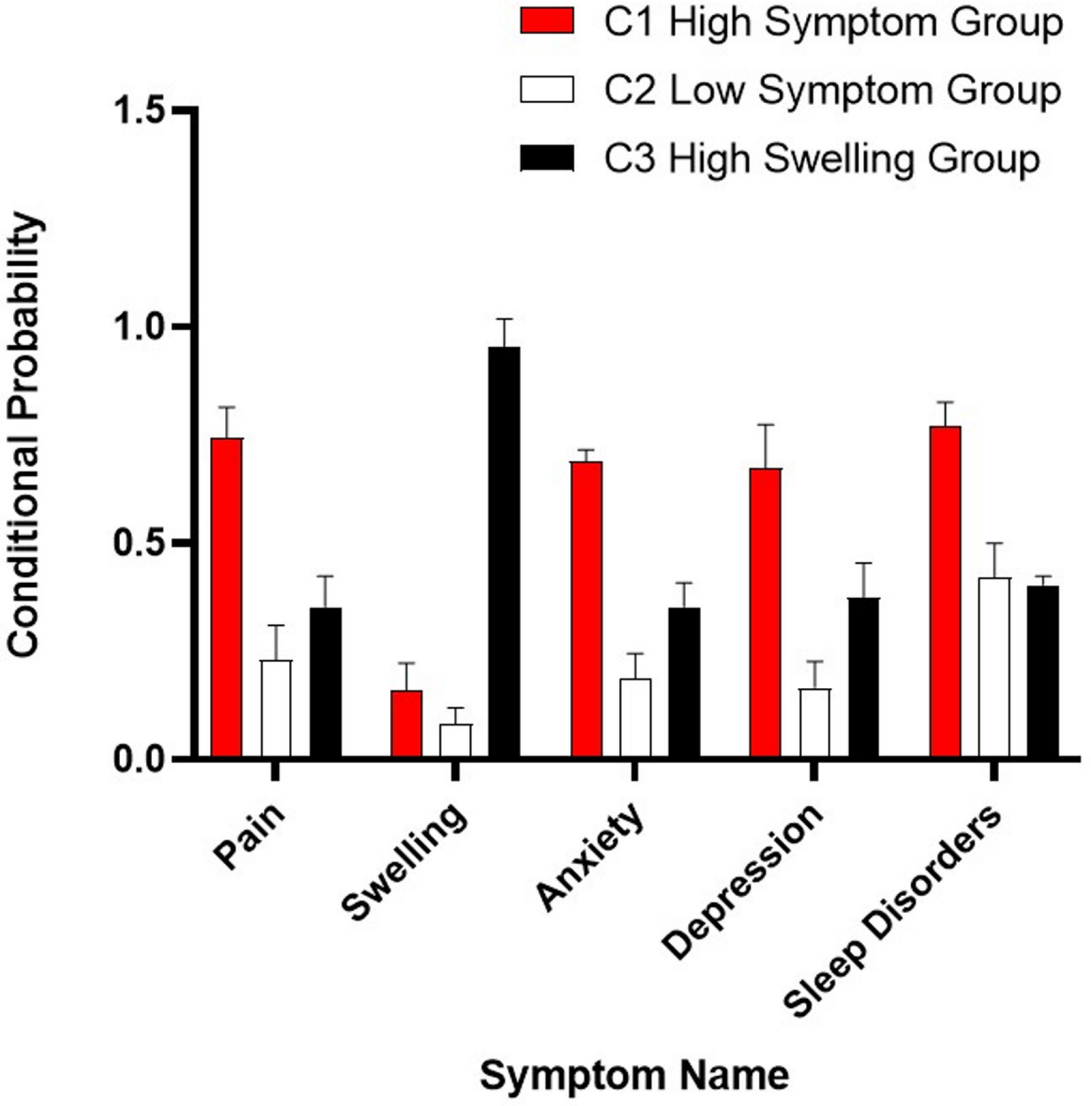
Latent class analysis characteristics of postoperative symptom clusters in KOA patients after TKA. KOA = knee osteoarthritis, TKA = total knee arthroplasty.

Figure 1 illustrates the results of the LCA performed to categorize postoperative symptom clusters in KOA patients following TKA. Three distinct symptom clusters were identified based on the patients’ responses to various postoperative symptoms, including pain, swelling, anxiety, depression, and sleep disorders.

The 3 identified latent classes are as follows:

Class 1 (hHigh symptom group): this group was characterized by severe postoperative symptoms, including high pain levels (VAS score), significant swelling, and elevated anxiety and depression scores (HADS). Patients in this cluster also reported poor sleep quality as assessed by the PSQI.

Class 2 (low symptom group): the low symptom group exhibited minimal symptoms across all categories, with lower pain, anxiety, and depression scores, and better sleep quality. This group represented the patients who experienced relatively mild postoperative discomfort and distress.

Class 3 (high swelling group): patients in this cluster displayed high levels of postoperative swelling, though their pain and psychological distress scores were moderate. Despite this, the high swelling group experienced significant physical symptoms that differentiated them from the low symptom group, particularly in terms of knee joint swelling.

The 3-class model was selected based on the fit indices (AIC, BIC, and aBIC) and an entropy of 0.825, indicating clear class separation.

### 3.2. Basic information of study patients and symptom cluster classification

This study included 365 KOA patients (93.8% of 389 valid respondents) with a mean age of 65.39 ± 7.1 years. Gender distribution revealed female predominance (75.9% vs 24.1% male). Postoperative symptom analysis demonstrated high prevalence rates: pain (98.0%, VAS 3.63 ± 2.02/10), swelling (89.3%, Likert 4.75 ± 2.81), anxiety (18.6%, HADS 4.75 ± 2.81), depression (22.7%, HADS 5.26 ± 2.83), and sleep disorders (46.0%, PSQI 7.98 ± 3.72; Table 1).

**Table 1:** Basic information of study patients and symptom cluster classification.

Item	Value
Total number of questionnaires issued	420
Valid returned questionnaires	389
Valid response rate	92.6%
Total number of patients	389
Proportion of patients with symptom clusters	365 (93.8%)
Average age of patients with symptom clusters gender	65.39 ± 7.1
Male	88 (24.1%)
Female	277 (75.9%)
Postoperative symptoms	
Pain	326 (89.3%)
Swelling	358 (98.0%)
Anxiety	68 (18.6%)
Depression	83 (22.7%)
Sleep disorders	168 (46.0%)
VAS score	3.63 ± 2.02
HADS anxiety score	4.75 ± 2.81
HADS depression score	5.26 ± 2.83
PSQI score	7.98 ± 3.72

HADS = Hospital Anxiety and Depression Scale, PSQI = Pittsburgh Sleep Quality Index, VAS = Visual Analog Scale.

### 3.3. Model fit indices for latent class analysis of postoperative symptom clusters

Table 2 displays the model fit indices for the LCA conducted to classify postoperative symptom clusters in KOA patients following TKA. Four models, each representing a different number of latent classes, were evaluated to determine the optimal number of symptom clusters. The 1-class model (AIC = 2087.136, BIC = 2105.311) did not adequately fit the data, as it failed to account for the variation in postoperative symptoms. The 2-class model (AIC = 2065.922, BIC = 2106.134) showed an improvement, but it still did not fully capture the heterogeneity of symptoms in the cohort. The 3-class model (AIC = 2053.092, BIC = 2117.285) demonstrated a better fit, with significantly improved indices and a clear distinction between the classes. Although the 4-class model (AIC = 2055.143, BIC = 2141.346) showed some improvement, it resulted in a more complex structure with smaller class probabilities that were harder to interpret. The Lo–Mendell–Rubin likelihood ratio test (LMRT) and bootstrap likelihood ratio test (BLRT) indicated that the 3-class model provided the best fit (*P* = .000), with the LMRT and BLRT indicated that the 3-class model provided the best fit (*P* < .001), with an entropy of 0.825, suggesting clear separation between the identified classes. The final model identified 3 distinct symptom clusters: the high symptom group (C1), characterized by severe symptoms across pain, anxiety, and sleep disturbances; the low symptom group (C2), with mild symptoms across all categories; and the high swelling group (C3), which was predominantly characterized by significant swelling, though with moderate pain and psychological distress. These findings indicate that the 3-class model best captures the complexity of postoperative symptom patterns in KOA patients after TKA, providing valuable insights into how these clusters might relate to recovery and quality of life outcomes.

**Table 2:** Model fit indices for latent class analysis of postoperative symptom clusters in KOA patients after TKA (n = 365).

Model	AIC	BIC	aBIC	*P* value		Entropy	Class probability
				LMRT	BLRT		
1	2087.136	2105.311	2093.445	–	–	–	1.000
2	2065.922	2106.134	2072.206	.026	.000	0.808	0.087/0.913
3	2053.092	2117.285	2076.213	.023	.000	0.825	0.162/0.516/0.324
4	2055.143	2141.346	2081.175	.197	.121	0.816	0.136/0.118/0.273/0.478

KOA refers to knee osteoarthritis, while TKA stands for total knee arthroplasty. AIC is the Akaike Information Criterion, BIC is the Bayesian Information Criterion, and aBIC is the sample-adjusted version of BIC. LMRT represents the Lo–Mendell–Rubin likelihood ratio test, and BLRT is the bootstrap-based likelihood ratio test. The dash (–) indicates a blank item.

### 3.4. Comparison of general information for potential symptom clusters after TKA surgery in KOA patients

Table 3 presents a comparison of demographic and clinical characteristics across the 3 identified symptom clusters (C1, C2, and C3) in patients following TKA for KOA. Significant differences were observed in several key variables, including age, BMI, disease duration, and the presence of chronic disease comorbidities. Age distribution differed significantly across the symptom clusters (χ^2^ = 9.963, *P* = .008), with the high symptom group (C1) having a larger proportion of patients aged 60 years or older compared to the low symptom group (C2). The high symptom group (C1) and high swelling group (C3) also had a higher prevalence of higher BMI categories (χ^2^ = 4.536, *P* = .021), with 47.4% and 52.5% of patients in these groups having a BMI ≥ 28.0 kg/m^2^, respectively, compared to only 33.8% in the low symptom group (C2). Disease duration was significantly longer in the high symptom and high swelling clusters, with a higher proportion of patients reporting symptoms for more than 5 years (χ^2^ = 5.466, *P* = .021). Chronic disease comorbidities were more prevalent in the high symptom (C1) and high swelling (C3) groups (χ^2^ = 7.468, *P* = .021), indicating a potential link between the presence of additional health conditions and the severity of postoperative symptoms. These findings suggest that factors such as older age, higher BMI, longer disease duration, and the presence of chronic comorbidities are associated with more severe symptom clusters after TKA.

**Table 3:** Comparison of general information for potential symptom clusters after TKA surgery in KOA patients (n, %).

Indicators	C1 (n = 59)	C2 (n = 186)	C3 (n = 120)	χ^2^/*H*	*P* value
Age (yr)					
45–59	25 (42.3)	46 (24.7)	20 (16.6)	χ^2^ = 9.963	.008
≥60	34 (47.6)	140 (75.2)	100 (83.3)		
Gender					
Male	10 (16.9)	42 (22.5)	36 (30.0)	χ^2^ = 4.776	.083
Female	49 (83.0)	144 (77.4)	84 (70.0)		
BMI					
<24.0	9 (15.2)	20 (10.7)	26 (21.6)	*H* = 13.342	.001
24.0–27.9	10 (16.9)	65 (34.9)	40 (33.3)		
≥28.0	40 (67.7)	101 (54.3)	54 (45.0)		
Living situation					
With spouse	32 (54.2)	108 (58.0)	72 (60.0)	χ^2^ = 4.536	0.566
With children/parents	16 (27.1)	48 (25.8)	28 (23.3)		
Living alone	6 (10.1)	20 (10.7)	11 (9.1)		
Other	5 (8.4)	10 (5.3)	9 (7.5)		
Marital status					
Married	45 (76.2)	143 (76.8)	88 (73.3)	χ^2^ = 2.584	.279
Divorced/widowed	14 (23.7)	43 (23.1)	32 (26.7)		
Education level					
Elementary or below middle school	21 (35.5)	82 (44.0)	55 (45.8)	H = 1.173	.556
	20 (33.8)	48 (25.8)	32 (26.6)		
High school	9 (15.2)	36 (19.3)	23 (19.1)		
College and above	9 (15.2)	20 (10.7)	10 (8.3)		
Occupation					
Employed/individual	8 (13.5)	38 (20.4)	10 (8.3)	χ^2^ = 3.736	.176
Farmer	25 (42.3)	55 (29.5)	45 (37.5)		
Unemployed/retired	20 (33.8)	60 (32.2)	50 (41.6)		
Other	6 (10.1)	33 (17.7)	15 (12.5)		
Smoking history					
Yes	28 (47.4)	85 (45.6)	63 (52.5)	χ^2^ = 0.573	.774
No	31 (52.5)	101 (54.3)	57 (47.5)		
Drinking history					
Yes	30 (50.8)	73 (39.2)	56 (46.6)	χ^2^ = 1.682	0.434
No	29 (49.1)	113 (60.7)	64 (53.3)		
Family monthly income (RMB)					
<2000	20 (33.8)	63 (33.8)	38 (31.6)	*H* = 1.473	.479
2000–3999	25 (42.3)	55 (29.5)	45 (37.5)		
4000-5999	10 (16.9)	43 (23.1)	20 (16.7)		
≥6000	4 (6.7)	25 (13.4)	17 (14.1)		
Disease duration (yr)					
≤5	25 (42.3)	58 (31.1)	53 (44.1)	χ^2^ = 5.466	.067
>5	34 (57.6)	128 (68.8)	67 (55.8)		
Chronic disease comorbidity					
Yes	35 (59.3)	106 (56.9)	68 (56.6)	χ^2^ = 0.193	.901
No	24 (40.6)	80 (43.0)	52 (43.3)		
Number of joint surgeries					
1	50 (84.7)	126 (67.7)	80 (66.7)		
≥2	9 (15.2)	60 (32.2)	40 (33.3)	χ^2^ = 7.468	.021
Surgical duration (h)					
<2	38 (64.4)	118 (63.4)	78 (65.0)	χ^2^ = 0.236	.884
≥2	21 (35.5)	68 (35.5)	42 (35.0)		
Intraoperative blood loss (mL)					
≤100	38 (64.4)	105 (56.4)	64 (53.3)	χ^2^ = 3.738	.158
>100	21 (35.5)	81 (43.5)	56 (46.7)		
Use of tourniquet during surgery					
Yes	50 (84.7)	168 (90.3)	105 (87.5)	χ^2^ = 0.911	.648
No	9 (15.2)	18 (9.6)	15 (12.5)		

RMB = Renminbi (Chinese Yuan).

### 3.5. Variable assignment

Table 4 outlines the variable assignments used in the analysis of symptom cluster classification and their association with clinical outcomes. The dependent variable in the analysis was the symptom cluster latent category, which was categorized into 3 groups: high symptom group (C1), low symptom group (C2), and high swelling group (C3). The independent variables included a range of demographic and clinical factors. For gender, male was coded as 1 and female as 2. Age was divided into 2 categories: patients aged 60 years or older were coded as 1, and those aged between 45 and 59 years were coded as 2. BMI was classified into 3 categories: underweight (<24.0 kg/m^2^), overweight (24.0–27.9 kg/m^2^), and obese (≥28.0 kg/m^2^), coded as 1, 2, and 3, respectively. The number of joint surgeries was coded as 1 for the first surgery and 2 for patients who had undergone 2 or more surgeries. Occupation was categorized into 4 groups: employed/self-employed, farmer, unemployed/retired, and other. Disease duration was classified as ≤5 years (coded as 1) and >5 years (coded as 2). Finally, intraoperative blood loss was classified as ≤100 mL (coded as 1) and>100 mL (coded as 2). These variable assignments were used in subsequent analyses, including multinomial logistic regression, to assess the associations between the identified symptom clusters and the clinical characteristics of the patients.

**Table 4:** Variable assignment.

Variable	Assignment
Dependent variable	
Symptom cluster latent category	1 represents high symptom group; 2 represents low symptom group; 3 represents high swelling group
Independent variables	
Gender	1 represents male; 2 represents female
Age	1 represents ≥60 yr; 2 represents 45–59 yr
BMI	1 represents <24.0 kg/m^2^; 2 represents 24.0 kg/m^2^; 3 represents ≥28.0 kg/m²
Number of joint surgeries	1 represents the first surgery; 2 represents ≥2 surgeries
Occupation	1 represents employed/self-employed; 2 represents farmer; 3 represents unemployed/retired; 4 represents other
Disease duration	1 represents ≤5 yr; 2 represents >5 yr
Intraoperative blood loss	1 represents ≤100 mL; 2 represents >100 mL

The dependent variable uses the low symptom group as the reference category; the independent variables use the category with the largest assignment as the reference.

BMI = body mass index.

### 3.6. Multivariate unordered multinomial logistic regression analysis of symptom cluster latent categories after TKA in KOA patients

Table 5 presents the results of the multivariate unordered multinomial logistic regression analysis used to assess the association between symptom cluster latent categories and clinical variables. The regression model included independent variables such as BMI, number of joint surgeries, and intraoperative blood loss, which were examined for their potential influence on the classification of patients into the high symptom group (C1) and high swelling group (C3), relative to the low symptom group (C2). Significant associations were found between BMI and both the high symptom and high swelling groups. Specifically, patients with a BMI between 24.0 and 27.9 kg/m^2^ had a significantly higher likelihood of being in the high symptom group (C1; OR = 2.736, 95% CI = 1.216–6.139, *P* = .018) and high swelling group (C3; OR = 2.773, 95% CI = 1.323–5.811, *P* = .015) compared to those with a BMI < 24.0 kg/m^2^. Similarly, patients undergoing their first joint surgery were significantly more likely to belong to the high-symptom group (C1; β = 1.006; OR = 2.735, 95% CI 1.323–5.811; *P* = .007) than to the low-symptom reference group. These findings suggest that a higher BMI and undergoing a first surgery may predispose patients to more severe postoperative symptoms, including pain, swelling, and psychological distress. The results underscore the importance of addressing obesity and preoperative conditions to better manage symptom clusters post-TKA and improve recovery outcomes for patients.

**Table 5:** Multivariate unordered multinomial logistic regression analysis of symptom cluster latent categories after TKA in KOA patients.

Variable	Partial regression coefficient	Standard error of regression coefficient	Wald χ^2^	*P* value	OR value	95% CI of OR value
High symptom group						
BMI 24.0–27.9 kg/m^2^	−1.018	0.425	5.603	.018	0.363	(0.155, 0.843)
First joint surgery	2.735	0.416	5.924	.015	2.736	(1.216, 6.139)
High swelling group						
BMI < 24.0 kg/m^2^	1.018	0.378	7.276	.007	2.773	(1.323, 5.811)

BMI = body mass index, CI = confidence interval, KOA = knee osteoarthritis, OR = odds ratio, TKA = total knee arthroplasty.

### 3.7. Comparison of SF-12 scores among different symptom cluster latent categories after TKA in KOA patients

Table 6 presents a comparison of the 12-Item Short Form Health Survey (SF-12) scores (both total and physical and mental dimension scores) across the 3 identified symptom clusters (C1, C2, and C3) in patients after TKA for KOA. The analysis revealed significant differences in the total SF-12 scores and in the mental health dimension scores between the clusters. The high symptom group (C1) showed significantly lower total SF-12 scores (50.38 ± 12.96) compared to both the low symptom group (C2; 56.63 ± 9.56) and the high swelling group (C3; 57.42 ± 10.85), with these differences reaching statistical significance (*F* = 6.428, *P* = .002). Additionally, the high symptom group (C1) had significantly lower mental health dimension scores (55.22 ± 13.72) compared to the low symptom group (C2; 63.17 ± 11.33) and the high swelling group (C3; 63.61 ± 12.23), with these differences also being statistically significant (*F* = 10.643, *P* < .001). However, no significant difference was found in the physical health dimension scores between the groups (*F* = 2.212, *P* = .111). These results highlight the considerable impact of symptom severity, particularly psychological symptoms, on the overall quality of life and mental health of patients post-TKA, suggesting that those in the high symptom group (C1) experience significantly worse mental well-being and overall life satisfaction.

**Table 6:** Comparison of SF-12 scores among different symptom cluster latent categories after TKA in KOA patients (*x* ± *s*, points).

Indicator	C1 (n = 59)	C2 (n = 186)	C3 (n = 120)	*F* value	*P* value
Total score	50.38 ± 12.96	56.63 ± 9.56	57.42 ± 10.85	6.428*	.002
Physical dimension	42.75 ± 16.97	46.38 ± 13.11	47.63 ± 14.07	2.212†	.111
Mental dimension	55.22 ± 13.72	63.17 ± 11.33	63.61 ± 12.23	10.643†	.000

C1 = high symptom group, C2 = low symptom group, C3 = high swelling group, KOA = knee osteoarthritis, SF-12 = 12-item short form health survey, TKA = total knee arthroplasty.

* Welch test.

† One-way analysis of variance.

### 3.8. Pairwise comparison of SF-12 scores among different symptom cluster latent categories after TKA in KOA patients

Table 7 presents the pairwise comparisons of SF-12 scores between the 3 symptom clusters identified after TKA in KOA patients. Significant differences were observed in both the total SF-12 scores and the mental health dimension scores across the clusters. The high symptom group (C1) had a significantly lower total SF-12 score compared to both the low symptom group (C2; MD = −6.28, 95% CI = −10.86 to −1.68, *P* = .003) and the high swelling group (C3; MD = −7.06, 95% CI = -11.96 to −2.13, *P* = .004), indicating that patients in the high symptom group reported a significantly worse overall quality of life. In terms of mental health, the high symptom group (C1) also had significantly lower scores compared to the low symptom group (C2; MD = −7.93, 95% CI = −11.57 to −4.32, *P* < .001) and the high swelling group (C3; MD = −8.45, 95% CI = −12.35 to −4.56, *P* < .001), suggesting that severe postoperative symptoms are associated with poorer mental health outcomes. Conversely, there were no significant differences in either the total SF-12 scores or the mental health scores between the low symptom group (C2) and high swelling group (C3). The total score difference between C2 and C3 for the total SF-12 score was minimal (MD = −0.78, 95% CI = −3.82 to 2.26, *P* = .903), and the mental health dimension showed no significant difference (MD = −0.49, 95% CI = −3.34 to 2.36, *P* = .736). These findings highlight the significant impact of symptom severity, particularly in the high symptom group (C1), on both physical and mental health outcomes, emphasizing the need for targeted postoperative interventions to improve quality of life in these patients.

**Table 7:** Pairwise comparison of SF-12 scores among different symptom cluster latent categories after TKA in KOA patients.

Indicator	C1 vs C2		C1 vs C3		C2 vsC3	
	MD 95% CI	*P* value	MD 95% CI	*P* value	MD 95% CI	*P* value
Total score	–6.28 (–10.86, –1.68)	.003	–7.06 (–11.96, –2.13)	.004	–0.78 (–3.82, 2.26)	.903
Mental dimension	–7.93 (–11.57, –4.32)	.000	–8.45 (–12.35, –4.56)	.000	–0.49 (–3.34, 2.36)	.736

C1 = high symptom group, C2 = low symptom group, C3 = high swelling group, CI = confidence interval, KOA = knee osteoarthritis, MD = mean difference, SF-12 = 12-item short form health survey, TKA = total knee arthroplasty.

## 4. Discussion

This study identified 3 distinct postoperative symptom clusters in KOA patients following TKA: the high symptom group (C1), the low symptom group (C2), and the high swelling group (C3). The high symptom group (C1) exhibited severe postoperative symptoms across pain, swelling, anxiety, depression, and sleep disturbances. The low symptom group (C2) showed minimal symptoms across all categories, with relatively mild pain and psychological distress. The high swelling group (C3), while similar to C1 in terms of swelling severity, presented moderate levels of pain and psychological symptoms, distinguishing it from the more severely affected high symptom group (C1). These symptom clusters differed significantly in terms of demographic and clinical characteristics. The high symptom group (C1) was more likely to be older, with a larger proportion of patients aged 60 or above, and had higher BMI values compared to the low symptom group (C2). Furthermore, patients in the high symptom group (C1) also reported longer disease duration and a higher prevalence of chronic comorbidities, such as hypertension and diabetes, than the other groups. These findings suggest that factors such as age, higher BMI, longer disease duration, and comorbidities may be associated with more severe postoperative symptoms and slower recovery. The presence of these characteristics in the high symptom group (C1) implies a need for more intensive postoperative management to address both the physical and psychological challenges these patients face, which could improve their recovery outcomes.

### 4.1. Comparison with previous studies

Similar to prior research, our study found that pain, swelling, and psychological distress (including anxiety and depression) are key factors that differentiate patients in the postoperative phase following TKA. Previous studies have often identified that patients who experience high levels of pain post-surgery tend to have a longer recovery period and a lower quality of life.^[22]^ Our high symptom group (C1), which exhibited the most severe pain and psychological symptoms, aligns with these findings, confirming the well-established link between pain and delayed recovery in TKA patients. However, 1 discrepancy with the existing literature lies in the categorization of swelling as a standalone distinguishing factor in the high swelling group (C3). While prior research often treats swelling as a secondary symptom to pain, this study highlights that swelling, when severe, can form a distinct symptom cluster with moderate psychological distress.^[23]^ This emphasizes the importance of considering swelling as a significant symptom in its own right, as opposed to merely an adjunct to pain or physical function. This is particularly important for patients who experience significant swelling but not as much pain, suggesting that swelling can be a unique barrier to recovery that requires separate management strategies. Previous studies have also emphasized the psychological impacts of TKA, showing that depression and anxiety can be common in the postoperative period and can significantly affect functional recovery.^[24]^ Our study supports this notion, especially with the high symptom group (C1) showing elevated scores in both anxiety and depression. Furthermore, the finding that the low symptom group (C2) experienced minimal psychological distress further underscores the critical role of mental health in post-TKA recovery. This study builds upon these findings by not only confirming the relationship between pain, swelling, and psychological distress but also by presenting a comprehensive analysis of how these symptoms interact to form distinct clusters. Unlike previous studies that primarily focus on isolated symptoms, our study’s use of LCA provides a more refined, multifaceted view of postoperative recovery. By classifying patients into distinct symptom clusters, we offer a deeper understanding of how different symptom patterns may predict recovery trajectories and quality of life. This approach allows for more personalized management of post-TKA care, which is a unique contribution to the field.

## 5. Study limitations

Several limitations should be acknowledged. First, although data were collected prospectively at multiple time points, the present association analysis was based on a single postoperative assessment (12 weeks), which limits inferences regarding the temporal evolution of symptom clusters; longitudinal trajectory modeling is warranted in future work. Second, the study was conducted at a single center, which may limit generalizability; multicenter cohorts with more diverse populations and surgical practices are needed to confirm our findings. Third, although adequate for the planned analyses, the sample size and the latent-class results remain dependent on the selected indicator variables and model specification; future studies should incorporate additional factors such as social support and lifestyle. Finally, because patients with prior knee surgery or major systemic conditions were excluded, the findings may apply primarily to patients undergoing primary TKA for moderate-to-severe KOA and may not generalize to revision arthroplasty or medically complex patients.

## 6. Conclusion

This study identified 3 distinct postoperative symptom clusters in KOA patients after TKA: the high symptom group (C1), low symptom group (C2), and high swelling group (C3). These clusters were associated with significant differences in demographic and clinical characteristics, such as age, BMI, and disease duration, which can influence recovery outcomes. By recognizing these symptom clusters, personalized treatment plans can be developed, addressing both physical and psychological aspects of recovery, ultimately improving patient care and outcomes. The findings highlight the importance of multidisciplinary approaches to manage the complex needs of TKA patients, though further research, particularly longitudinal studies, is needed to explore long-term effects and refine postoperative management strategies.

## Author contributions

**Conceptualization:** Lei Zhou.

**Data curation:** Lei Zhou.

**Formal analysis:** Lei Zhou.

**Investigation:** Xingren Chen.

**Methodology:** Xingren Chen, Tong Chen.

**Project administration:** Xingren Chen.

**Resources:** Xingren Chen, Qingxi Zhang, Tong Chen.

**Software:** Qingxi Zhang.

**Supervision:** Qingxi Zhang.

**Validation:** Qingxi Zhang, Tong Chen.

**Writing – original draft:** Lei Zhou.

**Writing – review & editing:** Lei Zhou.
